# New TNM staging in lung cancer (8^th^ edition) and future perspectives

**Published:** 2020-09-02

**Authors:** José-María Matilla, M. Zabaleta, E. Martínez-Téllez, J. Abal, A. Rodríguez-Fuster, J. Hernández-Hernández

**Affiliations:** ^1^Department of Thoracic Surgery, University Hospital of Valladolid, Valladolid, Spain; ^2^Department of Pneumonology, Valdecilla University Hospital, Santander, Spain; ^3^Department of Thoracic Surgery, Santa Creu y Sant Pau Hospital, Barcelona, Spain; ^4^Department of Pneumonology, University Hospital of Orense, Orense, Spain; ^5^Department of Thoracic Surgery, Hospital del Mar, Parc de Salut Mar. Mar Institute of Medical Research. Barcelona, Spain; ^6^Department of Pneumonology, Complejo Asistencial de Ávila, Ávila, Spain

**Keywords:** TNM classification, staging, lung cancer

## Abstract

**Background:**

Carrying out a correct anatomical classification of lung cancer is crucial to take clinical and therapeutic decisions in each patient.

**Aim:**

TNM staging classification provides an accurate anatomical description about the extension of the disease; however, the anatomical burden of the disease is just one aspect that changes the prognosis.

**Relevance for Patients:**

TNM staging classification is a tool that predicts survival, but we must consider that TNM is just one of the factors that concern the prognosis. The impact of a factor over the prognosis is complex due to: It depends on the specific environment, the treatment strategy, among others, and our level of certainty makes difficult to include all the factors just in a group of stages. In some groups, there are difficulties to get large series due to the low frequency of cases and the small number of events (metastasis, locoregional recurrence). It does not allow to obtain evidence in a short period of time. On the other hand, in the next years, new markers will be incorporated in the coming years, which are going to be included in the new TNM classification. It could help to improve the classification giving more information about prognosis and risk of recurrence. All these aspects are being used by the International Association for the Study of Lung Cancer (IASLC) to develop a new prognosis model. This continues the evolution of TNM system, allows us to overcome the difficulties, and build a flexible framework enough to continue improving the individual prognosis of the patients.

## 1. Introduction

Carrying out a correct anatomical classification of lung cancer is crucial to take clinical and therapeutic decisions in each patient [1]. The classification consists of three components: T (tumor) N (nodes) and M (metastases) in four stages (I to IV). This classification allows us to standardize the extension of lung tumors. It compares the clinical information collected from different centers with the purpose to homogenize patients in groups (stages) with similar prognosis and therapeutic approaches. The information related to the extension of the disease could be necessary too to decide if an individual could be part of a trial or not [2]. Lung cancer is unique among the different types of cancer with respect to stage classification. Most cancers staging systems are based on empirical results and based on consensus. The classification of lung cancer is the result of a sophisticated system of statistical analysis of more than 100,000 patients [3].

In 2017, the *Union Internationale Contre le Cancer* (UICC) later called *Union for International Cancer Control* and the *American Joint Committee for Cancer Staging and End Results Reporting (AJC)*, later known as *The American Joint Committee on Cancer* (AJCC), in collaboration with the *International Association for the Study of Lung Cancer* (IASLC) agreed to publish the 8^th^ edition of lung cancer TNM [4]. Although the classification in stages remains stable over the years, the AJCC and the UICC make regular reviews. The 8^th^ edition is a result of a multidisciplinary and continuous improvement of the previous ones. Following this idea, new patient data have been collected to develop the 9^th^ edition which is expected to be completed in 2024 [5].

## 2. The Beginnings

Although several international organizations worked on the staging classifications, the T, N, and M descriptors were proposed and developed by the French surgeon Pierre Denoix between 1943 and 1952 [6-8]. The first edition of the general classification of TNM tumors was published in 1968 under the supervision of the UICC. They included cases classified between T0 (no visible tumor) and T4 (tumors with the extension beyond the lung), from Nx to N1 (intrathoracic lymph nodes) and category M1 was subdivided into M1a (malignant pleural effusion), M1b (cervical nodes palpable), and M1c (distant metastases) [8].

In 1959, the AJC was created independent from the UICC. It made that the staging classification of both organizations was not exactly the same [9]. The lung cancer working group (Drs. Mountain, Carr, and Anderson) analyzed the characteristics and progress of 2155 patients. These studies were the basis to develop T and N, sections, and to introduce the idea of TNM stages, groups, or subsets for with similar prognosis. These proposals were included in the second TNM classification of the UICC (1975) being the first edition of the AJC cancer staging manual [9]. Fortunately, on the proposals of Dr. Mountain, an agreement was reached between the AJCC and the UICC bring their opinions closer, creating a common international classification in 1988 [8,9].

Until the first publication of the staging manuals based on the 6^th^ edition in 2002, the TNM updates were conducted by the M.D. Anderson Cancer Center in the United States of America (USA) created by Dr. Mountain [6,7]. In 1997, IASLC takes part in the review process for future classifications [1]. In that year, the 5^th^ edition was published, which had been based on the biggest series (5319 patients) so far of non-small cell lung cancers (NSCLC) [9]. The IASLC created, in the same year, a Staging Committee with international representation and supported by the non-profit medical-statistical organization *Cancer Research and Biostatistics* (CRAB) [9]. The first phase of the staging project collected a retrospective database of 81,495 evaluable records of patients with lung cancer (13,032 SCLC and 68,463 NSCLC), diagnosed between 1990 and the year 2000, including all therapeutic modalities. It was proposed to collect the cases contributed by 20 databases of patients with a follow-up for at least 5 years, also carrying out an internal (in different geographical areas) and external (using the *National Cancer Institute Surveillance, Epidemiology, and End Results Program* (SEER) database of the *American National Cancer Institute*) validation. The distribution of cases was: 58% came from Europe, 21% from North America, 14% from Asia, and 7% from Australia [9].

With this great project underway, and awaiting its results, the 6^th^ edition of the TNM was published in 2002, without any change over the 5^th^. The IASLC study conclusions were presented to the international community in 2007 and accepted by UICC and AJCC. This agreement was the origin of the 7^th^ edition of the TNM classification of lung cancer [10]. They described an accurate definition of visceral pleura involvement and they eliminate the differences between the two mediastinal lymph node maps used so far, Naruke map and Mountain/Dressler map [11].

Despite the great methodological and seriousness progress observed in the 7^th^ edition, there were improvements that justified further research. The initial retrospective project presented several limitations due to the lack of a specific database, for example, the changes proposed for the T descriptors was limited to the size, the existence of additional nodules or the presence of pleural effusion, and the lack of information for analyzing other factors such as tumor extension [3,12,13]. With the purpose of improving the database, they proposed to increase the number of non-surgical stages patients to get closer to the daily clinic and it was considered convenient a greater representation of other countries [3].

The second phase created to overcome the limitations of a retrospective project was the creation of a prospective database designed specifically for the TNM classification in lung cancer. Between 1999 and 2010 data, 94,708 patients were collected from four continents (Table 1).

**Table 1:** General characteristics of the databases used for the 7^th^ and 8th edition of the TNM classification of lung cancer.

General characteristics	7^th^ edition database	8^th^ edition database
Diagnostic period	1990-2000	1999-2010
Total of patients	100,869	94,708
Geographical origin		
Europe	58,701 (58%)	46,560 (49%)
North America	21,130 (21%)	4660 (5%)
Asia	11,622 (11,5%)	41,705 (44%)
Australia	9416 (9,3%)	1593 (1,7%)
South America	0	190 (0,3%)
Excluded patients	19,374 (19%)	17,552 (18%)
Treatment modalities		
Surgery +/- adjuvant treatment	50%	80,3%
Chemotherapy	23%	9,3%
Radiotherapy	11%	1,5%
Chemo + Radiotherapy	12%	4,7%
Trimodal treatment	3%	4,4%

The information collected in this new database includes general characteristics of the patient, diagnostic descriptors of the disease, various general laboratory parameters at the time of diagnosis, SUV (standardized uptake value) of Positron Emission Tomography (PET), and some results of the respiratory function. The evaluation of the clinical TNM was performed by collecting data related to radiological studies, CT and PET scan, bronchoscopy, and the different invasive diagnostic procedures, including surgical procedures with no tumor resection. In the treatment section, the TNM classification of each patient was collected according to the 7^th^ edition in force until that time. In the case of non-surgical patients, clinical TNM was used, and the type of treatment performed (chemotherapy and/or radiotherapy). In the case of patients treated surgically, the clinical TNM and pathological TNM were included, collecting different variables in relation to the surgical procedure, induction or adjuvant treatment, and the final pathological study. Information was also collected for special cases such as the presence of multiple nodules related to a unique tumor or the presence of synchronous tumors, being recorded separately in the latter case. Finally, follow-up data and treatment results were collected. In relation to obtaining more information for further studies, a few data were collected in order to identify potential resources for future projects (Table 2) [13].

**Table 2:** Variables collected for the 8^th^ edition and for the 9^th^ edition.

Variables		Variables database 8^th^ edition	News 9^th^ edition
General features		Age, race, sex, smoking, weight loss in the previous 6 months, performance status, specific weight, height, and comorbidities (Colinet score: Smoking, kidney failure, cardiovascular, respiratory, neoplastic comorbidities, alcoholism).	
Diagnostic descriptors		Inclusion date, detection method, diagnostic confirmation (histological or cytological), location of the primary tumor, degree of tumor differentiation, histological type.	
		Small cell tumors: Paraneoplastic syndromes and type.	
Laboratory variables		LDH, Hb, Ca, Na, ALP, ALB, white blood cell, neutrophils, and platelets count	
Lung function		FVC; FEV1	
PET		SUV values: Primary tumor and for lymph nodes.	
TNM pre-treatment descriptors.		Clinical characteristics of the tumor, size, and extension.	
		Carcinomatosis lymphangitis.	
		Nodal involvement evaluation confirmed by biopsy or cytology	
Treatment	Nonsurgical treatment	Clinical TNM.	Immunotherapy
		Chemotherapy, radiotherapy and radiation area.	
	Surgical treatment	Type, extent, and degree of resection (complete, incomplete, or uncertain).	STAS
		Adjuvant treatment with chemotherapy and/or radiotherapy. Clinical and pathologic TNM.	
		• T-component: Pleural extension, vascular invasion, fissure situation, lymphatic and perineural invasion, and cytology of pleural lavage.	
		• N-component: Number of nodes explored, number of positive nodes, extracapsular involvement. All by regions N3, N2, and N1.	
		• M-component: Findings that imply changes in the indication of radical surgical treatment.	
Multiple nodes secondary to primary tumor		Size, histology, and distance of the nodules in relation to the primary tumor.	
Synchronous multiple tumors		Staging data for each lesion will be collected separately and independently for each lesion.	
Follow-up Molecular parameters		Follow-up date, situation in relation to the tumor, relapse and date, date of exitus.	
			Genetic biomarkers, type of mutation, and detection technique. Type of sample. Protein alterations: Type, detection technique, sample type, antibody used.

*Clinical history and examinations – radiology, PET, bronchoscopy, necessary invasive, or surgical examinations that do not include therapeutic resection of the tumor. **The lymph node evaluation will be carried out following the node map published by the IASLC in 2009. PET: Positron emission tomography; LDH: Lactate dehydrogenase; Hb Hemoglobin; Ca: Calcium; NA: Sodium; ALP: Alkaline phosphatase; FVC: Forced vital capacity; FEV1: Forced expiratory volume in the 1^st^ second; SUV: Standardized uptake value; STAS: Presence of free cells in the alveolar space

The analysis of all this information has helped to provide the recommendations of the current 8^th^ edition of the TNM finally implemented in 2018 after being accepted by the UICC and the AJCC in 2017 [2]. The AJCC decided to defer the application of the 8^th^ edition in the USA to January 1, 2018, while the UICC (applicable to the rest of the world) started the implementation of the current edition on January 1, 2017. The decision of the AJCC was based on several debates with other relevant organizations: SEER, the Centers for Disease Control and Prevention (CDC), the College of American Pathologists, the National Comprehensive Cancer Network (NCCN), the National Cancer Data Base, and the Commission on Cancer [2].

We present below the most significant changes introduced in the 8^th^ edition of the TNM system/stages of lung cancer and their future perspectives (Table 3).

**Table 3:** Most relevant changes in 8^th^ edition TNM.

Descriptor	Major changes in 8^th^ edition TNM
T descriptor	Subdivisions T1–T2 with 1 cm increments till 5 cm T3–T4 new size criteria: 5–7 and >7 cm Subsolid lesion: Invasive – solid part is measured for T factor Specific stage classification adapted to new T categories
N descriptor	No changes
M descriptor	Subdivision extrathoracic metastases M1b only one extrathoracic metastases Mic multiple metastases

## 3. T, N, M Descriptors

The analysis of the T component was complex due to different descriptors were taken into account: Tumor size, endobronchial location, atelectasis/pneumonitis, and invasion of anatomical structures close to the lung. To evaluate the prognosis of T descriptor, tumors with or without lymph node involvement were evaluated considering complete or incomplete resection. All findings were consistent in the populations analyzed [14]. These analyses showed that every centimeter of the tumor is important. It demonstrated a significantly different prognosis with a new cut-off to separate T1 to T4. It was observed that tumors with a size higher than 5 cm (now T3) have a worse prognosis compared with previous editions of the TNM classification, and those with more than 7 cm (now T4) have a similar prognosis. Another important finding regarding the endobronchial tumors is that the most important prognosis factor is the invasion of the carina while the distance of the tumor in relation to the carina was not important. The same happened with atelectasis/total pneumonitis, atelectasis, or pneumonitis that affects the entire lung had the same prognosis as atelectasis/partial pneumonitis. In contrast, invasion of the diaphragm (a descriptor of T3 in the seventh edition, T4 in the eighth edition) had a worse prognosis than other T3 descriptors and similar T4 tumors. Finally, it was found that the mediastinal pleural invasion was rarely used as a descriptor [15]. The recommendations for T descriptors in the eighth edition are shown in Table 4: New categories based on tumor size, endobronchial location < 2 cm of carina, and total atelectasis/pneumonitis were staged toward below (from T3 to T2), and the invasion of the diaphragm was modified (from T3 to T4), while the invasion of the mediastinal pleura was eliminated as a descriptor.

**Table 4:** Changes in T-descriptor in 8^th^ edition of TNM compared with 7^th^ edition.

T component	7^th^ edition	8^th^ edition
0 cm (pure lepidic adenocarcinoma 3 cm total size)	T1a if ≤ 2 cm;	Tis (AIS)
	T1b if >2-3 cm	
≤0.5 cm, invasive size (lepidic predominant adenocarcinoma ≥3 cm total size)	T1a if ≤ 2 cm;	T1mi
	T1b if >2-3 cm	
≤1 cm	T1a	T1a
>1-2 cm	T1a	T1b
>2-3 cm	T1b	T1c
>3-4 cm	T2a	T2a
>4-5 cm	T2a	T2b
>5-6 cm	T2b	T3
>6-7 cm	T3	T4
Bronchus <2cm from carina	T3	T2
Total atelectasis/pneumonitis	T3	T2
Invasion of diaphragm	T3	T4
Invasion of mediastinal pleura	T3	-

Specific analyses of the visceral pleural invasion descriptor showed that the two types of invasion (PL1, the tumor invades beyond the elastic layer and PL2, the tumor invades the pleural surface) had different prognoses, associating PL2 with a worse prognosis [16]. Therefore, the invasion of the visceral pleura is not only a negative prognostic factor but also the precise evaluation of the invasion depth must be actively performed, further stratifying the prognosis subgroups (PL1 and PL2).

All these new categories in T descriptor are relevant because they help to divide the patients into new groups associated with changes in treatment models. In the 8^th^ edition persists the discussion about which is the most accurate method to measure the nodules [17]. Finally, the conclusion was to measure based on the pulmonary window on CT scan [18], but, in contrast, other studies such as NELSON, the methods are different and probably it makes difficult to apply in common clinical practice [18].

Another important change was how to measure partially solid or subsolid tumors. For subsolid nodules, suggestive of non-mucinous adenocarcinoma, it involves measuring only the solid part on the CT scan to determine the size of the tumor. It allows a radiological-pathological and clinical correlation since it corresponds to the invasive tumor part in the pathological study [19]. These data take special relevance for the pathological entities of adenocarcinoma, *adenocarcinoma in situ* (AIS) and *minimally invasive adenocarcinoma* (MIA) that were defined by a common working group of the IASLC, American Thoracic Society (ATS), and the European Respiratory Society (ERS), subsequently joined the histological classification of lung, pleura, thymus, and heart tumors of the World Health Organization (WHO) of 2015 [20]. In these partially solid lesions, it is also recommended to record the total tumor size for later analysis and comparison.

The TNM classification of lung cancer classifies the N descriptor according to the location of nodal involvement, unlike other cancers such as digestive tumors – which consider the number of affected regional nodes. The N0-N3 categories have remained unchanged since the 4th edition of the UICC in 1987; the involvement of the supraclavicular and scalene nodes previously was considered M1. The pathologic nodal stage is the most important prognostic factor in resected NSCLC. This prognostic value depends on the thoroughness of surgical nodal dissection [21]. The 8^th^ edition confirms the consistency of the N descriptor to establish groups with significant differences in overall survival between the four categories N0-N3, both in clinical and pathological staging [22]. The prognostic impact of nodal involvement is also determined by the intensity of tumor load expressed by the number of affected nodes (nN) [23,24]. In this sense, the IASLC carried out a prognosis analysis on the combination of pN categories with the number of node stations involved in those cases M0 of the database used for the revision of the 7^th^ edition. The impact was subdivided according to whether it was single or multiple stations. Survival at 5y of the subgroups was: 58% in cases pN1 with a single affected station (pN1a); 50% when they had several positive stations pN1 (pN1b); 52% in cases with solitary pN2 without affectation N1 (pN2a1); 41% in the group with solitary pN2 and simultaneous affectation pN1 (pN2a2); and 36% in cases with multiple pN2 (pN2b) [22]. Despite their prognostic value, these subdivisions were not included in the 8^th^ edition because they had only been contrasted with cases with pathological staging. The IASCL recommends recording both, the number of affected nodes and their location, for prospective information collection for the future [22].

For the upcoming 9^th^ edition of TNM, the Staging Committee and Prognostic Factors of the IASLC (SPFC-IASLC) proposes the following study objectives for nodal involvement [6]:

a) To revalidate the prognostic impact of state N.
b) To study the prognostic impact of nodal extension. Carrying out the study of single or multiple nodal involvements in the N1/N2 locations and assess the impact of the number of nodes affected. Several studies have shown that the number of involved nodes is an independent prognostic factor in patients with a complete resection [23,24] and that classification according to the number of nodes affected (nN) is a better prognostic factor than location-based (pN) [25,26].
c) The ratio of nodes affected. This ratio expresses the relationship between positive nodes over the total of resected nodes. A recent meta-analysis has found that a low ratio is associated with better overall survival [27]. However, it seems reasonable to think that its predictive value may be influenced by several factors, the location of the nodal station affects (N1 vs. N2/N3); the total number of resected nodes – the prognostic potential is likely to be different in one case with one positive node of four resected nodes than in another case with five positive out of twenty, although the ratio is the same in both cases –; and the type of nodal dissection performed (systematic or sampling) [27].
d) To study the prognostic value of node size. Patients with bulky pN2 nodes (diameter > 2 cm) have a worse prognosis than those with smaller nodes, although paradoxically, in the same study, patients with micrometastases were associated with a worse prognosis [28].
e) Impact of extracapsular involvement. Extracapsular involvement is associated with a worse prognosis, as well as tumors with a high degree of malignancy with vascular and lymphatic invasion [29].
f) To study the N3 prognostic value. In the 7^th^ edition, all N3 cases without distant disease were grouped as IIIB. The 8^th^ edition has separated this condition into two IIIB substations (T1a-c/T2a-b N3M0) and IIIC (T3, T4 N3M0). However, the IASLC database only included 2488 cases with clinical or pathological N3 involvement without distance extension [22]. Validation analysis of the IIIB/IIIC classification based on SEER database between 1988 and 2013 confirms a significant difference in overall survival between both categories. The 5-year survival of the N3 global cohort was 8.4%, 9.2% in Group IIIB, and 7.7% in Group IIIC [30].

In M descriptor, the 8^th^ edition of the TNM classification introduced some changes compared with to the 7^th^ (Table 3). In the M1a category, the overall survival (OS) in patients with intrathoracic metastases (pleural and malignant pericardial effusion, pleural and pericardial malignant nodules, and contralateral separate tumor nodules) showed a similar prognosis, so it was no necessary to modify the M1a descriptor [15].

However, in the analyses of extrathoracic metastasis, there were clinically relevant findings: (a) Overall survival of all metastatic site locations was not significantly different; (b) single extrathoracic metastases had a significantly better prognosis than multiple extrathoracic metastases, although similar to intrathoracic metastases; and (c) multiple extrathoracic metastases in one or different organs had a similar prognosis. Following these findings, category M1b was redefined to include a single extrathoracic metastasis, and the new category M1c was created to include multiple extrathoracic metastases in one or different sites/organs. The M1a and M1b categories have a very similar prognosis but represent different types of anatomical spread. It makes sense, then, to keep them in different M-categories, but they are grouped together in the new stage IVa because they have a better prognosis than the M1c category (stage IVb) [31,32].

There are some special situations that require attention. In the *diffuse pulmonary adenocarcinoma type pneumonia*-like, if there is a multifocal disease, the classification of the tumor is based on the location of the areas involved: T3 if it is in the same lobe, T4 if it is in other ipsilateral lobes, and M1a if in the contralateral lung [33]. Moreover, in SCLC, the IASLC recommends that the subdivision of the M-descriptors into M1a, M1b, and M1c has to be the same as in the NSCLC. The evaluation of patients with M1b disease showed no significant differences in survival between patients with metastatic disease in a single organ or multiple organs. However, a significant difference in OS in the 1^st^ year was observed between patients with single-organ metastases when involving the brain and those with single or multiple metastases in other organs (36% vs. 23% and 20%, respectively) [34].

After the analyses made for the 8^th^ edition, there are some aspects of the M-component that needs further clarification. Thus, Dias *et al*. [35] validated in their study the classification proposed in the 8^th^ edition, but they also found that patients with single metastases had a significantly better prognosis than those with multiple metastases in one or more organs. In addition, at different locations, most had a similar prognosis, although adrenal metastases tended to have a worse prognosis. Similar findings observed by other authors [36], although with the limitation that they are all retrospective studies. However, there was sufficient data to create the new M1c category for tumors with multiple extrathoracic metastases in one or several organs.

Furthermore, the II Cooperative Group of Bronchogenic Carcinoma of SEPAR [37] in the 7^th^ edition assessed prospectively the different prognostic between the categories M1a and M1b. In the M1a subgroup, patients with pleural spread had a worse OS than those with contralateral nodule(s) (32 vs. 50 weeks), while single versus multiple nodules did not differ. Regarding to the M1b subgroup, patients with isolated metastases had significantly better OS than those with multiple sites/organs of metastases; and in cases with isolated metastases, those with single lesions were better off than with multiple lesions. However, only 7.9% of cases with contralateral pulmonary nodules were confirmed cytohistologically. The results support the division of category M1 into two subgroups, M1a and M1b. M1b could be divided in three categories: M1b1 (isolated metastases and only one lesion), M1b2 (multiple metastases in one organ), and M1b3 (multiple metastases at different sites). The OS was different between the subgroups (better in M1b1) [37].

In recent years, the staging in lung cancer has improved according to the developed-in imaging and invasive techniques. This improvement, together with the advances in molecular medicine [38], is going to be basic for the upcoming TNM classification. They are going to play an important role to analyze the ways to metastasize the tumor, treatment response, and survival [39].

For the 9^th^ edition of the TNM classification, the IASLC is collecting data of M1a category: Pleural and pericardial effusion, pleural and pericardial nodules, location and number of contralateral metastatic lesions. In category M1b, the site or organ affected, and in M1c, the number of lesions in an individual organ or multiple sites/organs. Single metastases and their size should be confirmed in the final pathological diagnosis for future results. All these data will help to better define oligometastatic and polymetastatic disease, which, together with the analysis of biomarkers, it will improve our prognostic capacity and maybe for therapy [5].

## 4. Other Factors

There are other factors apart from the anatomical description of the extension of the disease that affects the prognosis. This group of factors includes: Related to the patient (e.g., age and comorbidities), related to the tumor (e.g., histotype and molecular characteristics), related to the environment (e.g., access to care and geographical region), and related to the treatment (e.g., treatment received, quality of care, and response to treatment) [2]. In cases of more aggressive tumors, the prognosis is basically determined by the anatomical extension of the tumor [2]. In contrast, in less aggressive or less advanced tumors, prognosis will be determined by other factors such as health, age, the effectiveness of the treatment performed, health system, socioeconomic level, adherence to treatment, cultural aspects ….

One of the characteristics of tumors is the presence of mutations in the advanced disease that makes them a candidate to target therapies. Taking into account the data collected in the database, the Molecular Subcommittee – SPFC is trying to incorporate certain genetic mutations such as ROS1, ALK, EGFR, KRAS, HER2, and among others, which are quite characteristic. There is also a group of different EGFR mutations that could correspond to disease stages and could be included in the future. Other markers: The presence of a histologic pattern such as micropapillary, tumor interactions with the PDL1 host, differentiation considered as general biological aggressiveness, PET activity, or Ki-67 staining may be useful in establishing a structure showing a clear prognostic significance. All these molecular characteristics should be considered for inclusion in the staging classification by the SPFC-IASLC.

On the other hand, incomplete resection, currently defined as a residual disease in the resection margin [40], is associated with a high risk of recurrence, progression, and mortality, compared to complete resection (R0) [41,42]. To address this problem, the IASLC proposed a broader definition of incomplete resection (R reclassification), including the creation of a new category of uncertain resections with negative margins but a high risk of residual disease [43]. Therefore, these proposals need to be validated in the next edition of the TNM classification, by the new Residual Disease (R) Subcommittee. Uncertain resection due to suboptimal nodal evaluation is significantly the most common, increasing the risk of recurrence after resection with curative intent. This point is particularly important as it can change the comparison between lung cancer survival data according to the environment [22,44]. There are other variables that define this potential incomplete resection: Margin involvement, extracapsular extension, and less frequent positive cytology of the pleural or pericardial fluid [45]. Some authors already propose to consider the subcategorization of uncertainty R marking particularly adverse patient groups, for their involvement in lung cancer survival comparisons [45].

Moreover, in the next years, the progressive introduction of screening programs allows diagnosing the presence of small lung nodules. The consensus of the IASLC Strategic Screening Advisory Committee recommends anatomical sublobar resections for pure ground-glass opacity lesions (GGO) or with a solid part <2 cm located in the external third of the lung, after histological confirmation of T1aN0M0 status [46]. It also recommends a cytological analysis of the margins of resection. There are two clinical trials studying the role of sublobar resection in small tumors, CALGB 140503 (USA) y JCOG 0802 (Japan), which will provide more reliable data when the results are available [47]. Their results may modify the definition of complete resection.

As regards the Neuroendocrine Tumors Subcommittee, carcinoid tumors remained excluded from the AJCC staging manual, although many experts applied the TNM classification and its descriptors to these patients, being proposed their incorporation from the 7^th^ edition [48]. However, this inclusion is limited due to their odd structure, morphology, immunohistochemistry, and molecular characteristics [49]. In future editions, the survival curves keep overlapping between the different substations due to their better prognosis [49,50]. We have recently attended the publication of several articles that try to validate the TNM classification for bronchial carcinoids [49,51], talking about the possibility of modification of the TNM system [49,52]. Some studies show that despite the improvements introduced in the 8^th^ edition, this is still an imperfect system for lung carcinoid tumors, especially related to the tumor recurrence in Stages II and III [50,51]. The inclusion of carcinoid tumors in the TNM staging system has been the objective of several studies to highlight the importance of nodal involvement in the prognosis of these tumors [53,54], in opposition to tumor size which is not a significant variable in this type of tumors [49,55]. Other studies have been focused on the histological grade, analyzing the mitotic index as a predictor prognosis [55]. There are differences in DSS (disease-specific survival) according to the M subcategory. The DSS M1a patients are close to M0 patients with a non-significant Hazard Ratio (HR), and being better than M1b [50]. Cattoni *et al*. [49] propose to combine the pathological T-descriptor of the TNM classification and the histological type, trying to create a specific prognostic model. There are other factors that may provide information about recurrence possibilities such as Ki-67 [56,57]. The most important limitation to create a TNM for carcinoid tumors is the low number of cases introduced in the database [56], although this cannot imply the creation of a staging system specific to each tumor histotype.

The data collected to create the 8^th^ edition represents patients with lung cancer diagnosis between 1999 and 2010 being possible to draw a 5-year survival. There are differences in outcomes depending on the region and the specific treatment approaches, showing an improvement (30-50%) [58]. It could be necessary to validate the data collected by an outside database. However, an outside database does not allow to match all the data collected. Although there are data collected in recent years, such as in the SEER database, it is impossible to identify and reclassify all cases such as those with diaphragmatic invasion classified as T3 in the 7^th^ edition [32]. On the other hand, the decision of the AJCC to delay the implementation of the 8^th^ edition creates differences between the data collected in 2017 in the USA comparing with the rest of the world [2].

There are studies that have tried to validate the classification, although they do not find differences between Group IIA and IIB in the 8^th^ edition. These findings are in line with other validation attempts of the 7^th^ edition [59,60] and 8^th^ edition [61,62]. It demonstrates that the difference between the IIA and IIB stages is not as big as other groups [62], although these analyses are retrospective with a small number of patients from one center [60-62]. Therefore, it is necessary to restrict the databases and create national registries that can reduce the variability between areas being a strong help to improve the treatment of patients. In any case, stratification, according to the 8^th^ edition, is valid from the prognosis in patients with complete resection. Most studies conclude that the 8^th^ edition shows an improvement to differentiate between subgroups being an independent predictor for prognosis [62-64]. Therefore, the 8^th^ edition is superior in terms of survival and recurrence-free interval [62].

## 5. Conclusion

The 8^th^ and the previous editions of the TNM classification of lung cancer provide new categories, especially in T and M descriptors [1] (Table 5). Staging is a tool that defines survival, but it must be considered in conjunction with other factors that influence prognosis. The impact of a factor is complex as it depends on the specific environment, treatment strategy, and other aspects; and the level of certainty of our knowledge makes it difficult to find the ability to fit it into a real prognosis simply grouping by stages (Table 5) [65]. It is difficult to obtain a large series in a short period of time, especially in some groups, due to the low incidence and number of events (metastases, local recurrence). Moreover, new descriptors will be included in future editions and it will improve the classification in relation to its prognosis and risk of recurrence. All these data collected could be the key to create a prognostic model by the IASLC. This model could provide a better analysis of the individual prognosis [66].

**Table 5:** Lung cancer staging groups. Adapted from Detterbeck F [2].

Descriptor	Subcategory	N0	N1	N2	N3
T1 M0	T1a	IA1	IIB	IIIA	IIIB
	T1b	IA2	IIB	IIIA	IIIB
	T1c	IA3	IIB	IIIA	IIIB
T2 M0	T2a	IB	IIB	IIIA	IIIB
	T2b	IIA	IIB	IIIA	IIIB
T3 M0	T3	IIB	IIIA	IIIB	IIIC
T4 M0	T4	IIIA	IIIA	IIIB	IIIC
Any T M1	M1a	IVA	IVA	IVA	IVA
	M1b	IVA	IVA	IVA	IVA
	M1c	IVB	IVB	IVB	IVB
